# Unpredictable Sound Stress Model Causes Migraine-Like Behaviors in Mice With Sexual Dimorphism

**DOI:** 10.3389/fphar.2022.911105

**Published:** 2022-06-16

**Authors:** Fernanda Tibolla Viero, Patrícia Rodrigues, Julia Maria Frare, Náthaly Andrighetto Ruviaro Da Silva, Marcella de Amorim Ferreira, Ana Merian Da Silva, Gabriele Cheiran Pereira, Juliano Ferreira, Micheli Mainardi Pillat, Guilherme Vargas Bocchi, Romina Nassini, Pierangelo Geppetti, Gabriela Trevisan

**Affiliations:** ^1^ Graduate Program in Pharmacology, Federal University of Santa Maria (UFSM), Santa Maria, Brazil; ^2^ Graduate Program in Pharmacology, Federal University of Santa Catarina (UFSC), Florianópolis, Brazil; ^3^ Department of Health Science, Clinical Pharmacology and Oncology, University of Florence (UNIFI), Florence, Italy

**Keywords:** headache, nociception, CGRP, sex dichotomy, IL-6, TNF-α

## Abstract

Migraine represents one of the major causes of disability worldwide and is more prevalent in women; it is also related to anxiety symptoms. Stress, such as sound stress, is a frequently reported trigger in migraine patients, but the underlying mechanisms are not fully understood. However, it is known that patients with migraine have higher levels of plasma inflammatory cytokines and calcitonin gene-related peptide (CGRP). Stress mediated by unpredictable sound is already used as a model of painful sensitization, but migraine-like behaviors and sexual dimorphism have not yet been evaluated. This study characterized nociception and anxiety-related symptoms after the induction of sound stress in mice. C57BL/6 mice (20–30 g) were exposed to unpredictable sound stress for 3 days, nonconsecutive days. We observed enhanced plasma corticosterone levels on day 1 after stress induction. First, 7 days after the last stress session, mice developed hind paw and periorbital mechanical allodynia, grimacing pain behavior, anxiety-like symptoms, and reduced exploratory behavior. The nociceptive and behavioral alterations detected in this model were mostly shown in female stressed mice at day 7 post-stress. In addition, on day 7 post-stress nociception, these behaviors were consistently abolished by the CGRP receptor antagonist olcegepant (BIBN4096BS, 100 mg/kg by intraperitoneal route) in female and male stressed mice. We also demonstrated an increase in interleukine-6 (IL-6), tumor necrosis factor (TNF-α), and CGRP levels in stressed mice plasma, with female mice showing higher levels compared to male mice. This stress paradigm allows further preclinical investigation of mechanisms contributing to migraine-inducing pain.

## Highlights

• female mice presented increased nociception and anxiety-like symptoms after stress induction than male mice.
• plasma levels of inflammatory cytokines (IL-6 and TNF-α) and CGRP were increased in ST mice, particularly in the females.
• CGRP receptor antagonist olcegepant (BIBN4096BS) reduced nociception and anxiety-like symptoms in stressed mice.

## 1 Introduction

Migraine has a major prevalence in females under 50 years of age and has a challenging treatment (Gazerani and Cairns, 2020). It has also been shown that migraine may be related to psychiatric disorders, including anxiety (Karimi et al., 2021). Migraine attacks are elicited by a variety of agents, including stress induction (Kelman, 2007). Stress is the most common trigger reported in migraine patients; it can also increase the duration of the crisis and contributes to chronic migraine development (Kelman, 2007; Viero et al., 2022a).

Thus, different stress models have been used to induce migraine-like behaviors (periorbital/hind paw mechanical allodynia, grimacing pain behavior, and anxiety-related symptoms) in mice to better study the mechanisms involved in this painful disease. Previous studies have used acute or chronic stress caused by repetitive restrain stress paradigm, social defeat, chronic variable, and early life stress (Kaufmann and Brennan, 2018; Avona et al., 2020a; Eller et al., 2021). In addition, sound stress seems to be a relevant mediator of headache induction in patients (Ishikawa et al., 2019). Previous studies using an unpredictable sound stress model have observed the development of plantar nociception (hind paw allodynia and chemical hyperalgesia) in male rats (Khasar et al., 2009). Nevertheless, the development of migraine-like behaviors using this stress model has not yet been evaluated.

The relationship between calcitonin gene-related peptide (CGRP) signaling and stress-mediated migraine-like behaviors has been investigated in only one preclinical study (Avona et al., 2020a). CGRP has been implicated in the pathology of migraine for several decades (Khan et al., 2019), and recent clinical studies have further confirmed CGRP as a protagonist in migraine, using CGRP signaling inhibitors (Halker Singh et al., 2019). A CGRP antagonist (olcegepant, BIBN4096BS) was effective in reducing periorbital allodynia in different models of migraine-like pain (Dalenogare et al., 2021). Moreover, a CGRP injection to the trigeminovascular system caused periorbital mechanical allodynia (PMA) and anxiety-like behavior, which was prolonged in female rats (Araya et al., 2020).

CGRP may lead to the release of pro-inflammatory cytokines (Ray et al., 2021), and the levels of interleukine-6 (IL-6) and tumor necrosis factor alpha (TNF-α) were increased in the plasm of migraine patients (Ray et al., 2021). In fact, the application of IL-6 in the dura mater and cisterna magna causes both periorbital and hind paw cutaneous hypersensitivity (Avona et al., 2021). An injection of TNF-α also sensitized the dural meningeal nociceptors (Zhang et al., 2011). Previously, skeletal muscle hyperalgesia caused by an unpredictable sound stress model was reduced by antisense treatment targeting the IL-6 or TNF-α receptors (Dina et al., 2011).

Thus, the mechanisms involved in the stress induction of migraine-related pain need to be studied to establish a better treatment for this painful disease. Here, we initially characterized the periorbital/hind paw mechanical allodynia, grimacing pain behavior, and anxiety-related symptoms. Secondly, we detected the plasma levels of pro-inflammatory cytokines and CGRP, as well as the antinociceptive effect of a CGRP antagonist, in male and female mice after the induction of unpredictable sound stress.

## 2 Methods

### 2.1 Animals

Male and female adult C57BL/6 mice (20–30 g) were maintained in a humidity-controlled (55–65%) and temperature-controlled room (20–22°C) on a 12-h light/dark cycle, with food (pelleted form) and water *ad libitum*. Animals were housed eight per cage, with nesting material. All experiments were carried out in the light phase (between 7:00 a.m. and 7:00 p.m.), and the animals were acclimatized to the laboratory room for at least 1 h before the experiments. The protocols employed in our study were approved by the Institutional Committee for Animal Care and Use of the Federal University of Santa Maria (UFSM; #9818180820).

The experimental protocols followed the guidelines for Animal Research Reporting *In Vivo* Experiments (ARRIVE) (McGrath and Lilley, 2015). The experiments were also performed using the current ethical guidelines for the investigation of experimental pain in conscious animals, and the number of animals and the intensity of the noxious stimuli were the minimum necessary to demonstrate the consistent effects of the treatments. A group size of eight animals was determined for the behavioral experiments, using G∗Power (v3.1).

**FIGURE 1:**
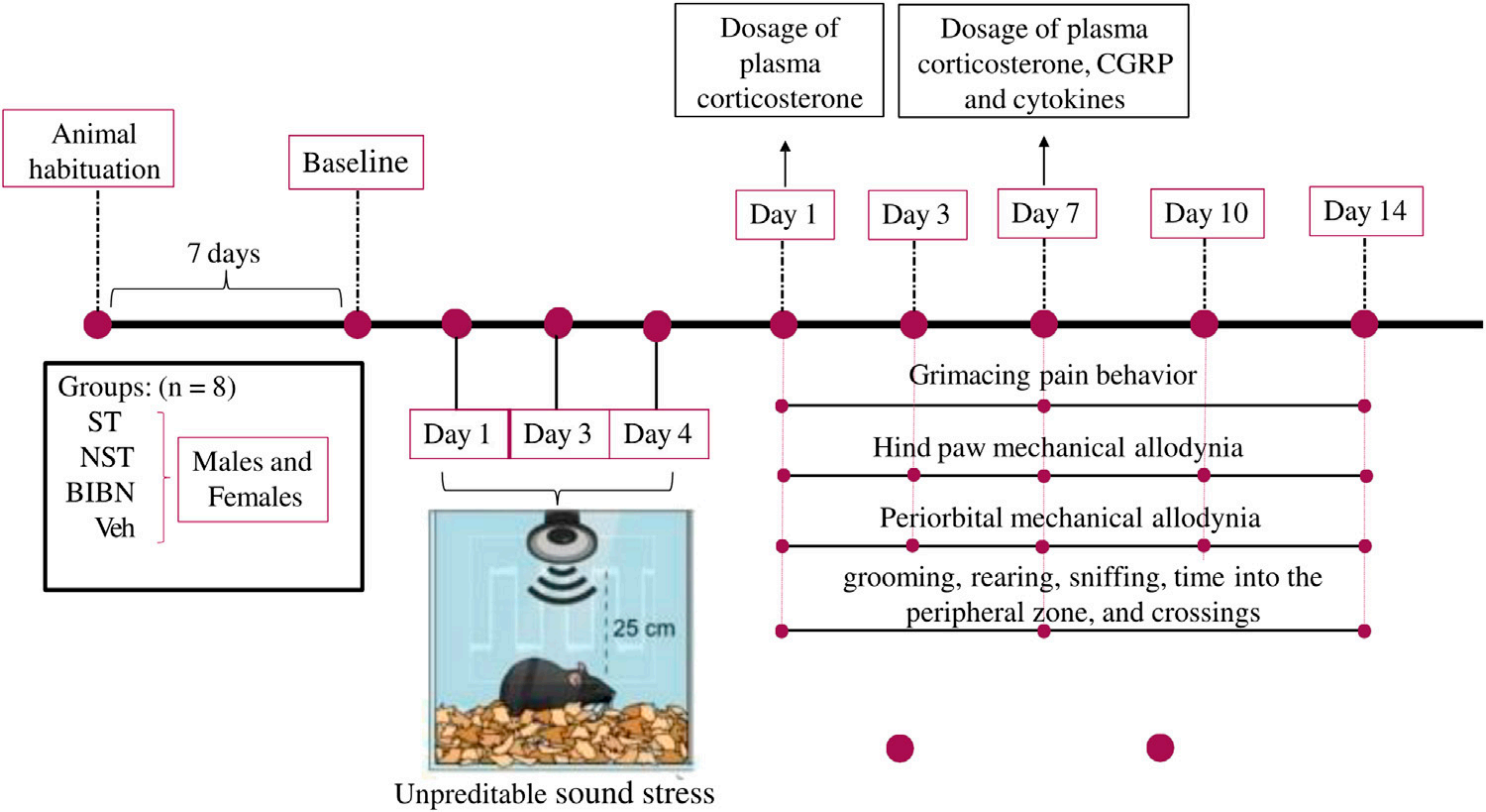
Schematic representation of experimental design. Exposure to unpredictable sound stress occurred over 3 days lasting for five or 10 s. Animals were exposed to the stressor for 3 nonconsecutive days (1, 3, and 4 days), for 30 min in each day. Day 1 post-stress induction described in our study, corresponds to day 5 (considering the beginning of the test). Animals were placed 4–5 per cage and the cage was placed 25 cm from a speaker that emitted four pure tones (5, 11, 15, and 19 kHz), whose amplitudes varied through time independently from 20–110 dB sound pressure level at random times each minute, lasting 5 or 10 s, and this unpredictable sound stress induction occurs for 30 min. Non-stressed (NST) animals were placed in the sound chamber for 30 min but without exposure to the sound stimulus. Following sound or non-stress, mice were returned to their home cages in the animal care facility. First, baseline grimace scale, periorbital and hind paw thresholds to von Frey filaments were recorded for each animal (7 days after animal habituation). After the last stress section induction (on day 4), the behavioral experiments were evaluated on days 1, 3, 7, 10, and 14 days. Subsequently, on the nociceptive peak after stress induction (7 days), the treatment protocol with CGRP antagonist and the different plasma analyses were performed (in a different group of animals).

All measurements of animal behavior were performed by the same researcher, who was blinded to the drug administration and to the group to be tested. The ST (stressed) and NST (non-stressed) groups (both female and male) were divided and studied on the same day, and experiments were replicated on different days, to generate results from the required number of mice (Figure 1, schematic representation of experimental design).

### 2.2 Reagents

All experimental reagents, if not specified in the text, were acquired from Sigma-Aldrich Chemical Co. (St. Louis, MO, United States), including BIBN4096BS (Catalogue No. SML2426).

### 2.3 Stress Induction

Animals were handled and acclimatized to the researcher and to all apparatus for 3 days before testing to reduce anxiety-like symptoms or stress. The exposure to unpredictable sound stress occurred over 3 days, as described previously (Khasar et al., 2008; Khasar et al., 2009). NST animals were placed in the sound chamber for 30 min, but without exposure to the sound stimulus on the same days as the stressed group. Animals were placed 4–5 per cage, and the cage was placed 25 cm from a speaker that emitted four pure tones (5, 11, 15, and 19 kHz), whose amplitudes vary independently through time, from 20–110 dB sound pressure level at random times each minute, lasting for five or 10 s. Animals were exposed to the stressor for three nonconsecutive days (Kelman, 2007; Kelman, 2007; Gazerani and Cairns, 2020; Viero et al., 2022a), for 30 min each day. Day 1 post-stress induction described in our study, corresponds to day 5 (considering the beginning of the test). Following sound or non-stress, the mice were returned to their home cages in the animal care facility. We did not exclude any animal from the study, and all animals were sensitized to this stress protocol, as described before (Khasar et al., 2008; Khasar et al., 2009).

### 2.4 Treatment Protocol

C57BL/6 J mice were injected intraperitoneally (i.p.) unilaterally on the right side. The injection was performed as quickly as possible by a single operator, with only minimal animal restraint. Eight female and male mice were treated with BIBN4096BS (i.p. 100 mg/kg) or its vehicle (4% dimethyl sulphoxide, DMSO, and 4% Tween 80 in 0.9% NaCl) (Burgos-Vega et al., 2019). Then, a total of 96 animals were used in the following animal grouping: 16 animals for the ST group (8 female and 8 male) and 16 animals for the NST group (8 female and 8 male). Second, the animals were divided into 8 groups (containing 8 animals for each sex): 1) ST + BIBN4096BS (male or female); 2) NST + BIBN4096BS (male or female); 3) ST + Veh male (male or female); and 4) NST + Veh (male or female).

### 2.5 Behavioral Experiments

Firstly, the baseline grimace scale and the periorbital and hind paw thresholds to von Frey filaments were recorded for each animal. Once the periorbital thresholds had been tested, animals were placed back in the hind paw testing chambers to habituate for approximately 5 min before beginning the hind paw testing (Tonello et al., 2014). After stress induction, the behavioral experiments were evaluated on days 1, 3, 7, 10, and 14. Subsequently, on the nociceptive peak after stress induction (7 days), the treatment protocol with CGRP antagonist, or the different plasma analyses, were performed.

### 2.6 Nociceptive Tests

The tests were carried out in a sequence, from the less stressful to the most stress-inducing test, as follows: grimace, hind paw mechanical allodynia, PMA, and then the open field test. All nociceptive measurements were performed on the same animal to reduce the number of animals used in the study.

#### 2.6.1 Evaluation of Grimacing Pain Behavior

The grimace scale quantifies changes in a few “action units” for mice, including orbital tightening, nose-cheek bulge, whisker tightening, and ear position Langford et al., 2010. Animals were placed in a Plexiglas chamber (5 × 5) of the von Frey test for 60 min. During this time, one image was captured every 2 min. The face images were then screened, labeled, randomly scrambled, and scored, with the researcher blinded to the treatment groups and the identity of each image. Overall, 30 images were blindly selected for each animal (per treatment condition or time-point), and each action unit on each image was given a score of 0, 1, or 2, as previously described (Dixon, 1980). The mean grimace scores were calculated as the average score across all the action units. This behavior scale was evaluated on days 1, 7, and 14 post-stress induction or in the NST animals. Grimace was also measured before ST or NST induction (basal), after ST or NST induction (7 days after), and 1 and 3 h after BIBN4096BS/vehicle treatment.

#### 2.6.2 Hind Paw Mechanical Allodynia

To evaluate the development of mechanical allodynia, the mice were individually placed in transparent boxes on a wire mesh platform that allowed easy access to the right hind paw plantar surface. Filaments of different stiffness were applied to the plantar surface of the hind paw, ranging from 0.07 to 2.0 g (0.07, 0.16, 0.40, 0.60, 1.0, 1.4, and 2.0 g). The mechanical threshold was obtained according to the up-and-down paradigm (Tonello et al., 2014).

This paradigm continued for a total of six measurements, or until four consecutive positive or four consecutive negative responses occurred. The mechanical paw-withdrawal threshold response (in g) was then calculated from the resulting scores (Dixon, 1980). To determine the baseline thresholds, the animals were acclimatized for 60 min before the test, and all animals were assessed before stress induction (baseline values). The mechanical threshold was evaluated on days 1, 3, 7, 10, and 14, and the hind paw mechanical allodynia was also measured before ST or NST induction (basal), after ST or NST induction (7 days after), and 1 and 4 h after BIBN4096BS/vehicle treatment.

#### 2.6.3 Periorbital Mechanical Allodynia

The measurement of PMA was performed using the up-and-down paradigm, as described previously (Dixon, 1980). Animals were allocated in a restraint apparatus designed for the evaluation of periorbital mechanical thresholds. The apparatus consisted of an individual clear three-walled plexiglass box (5 × 5) with an opening for the tail and one for the head and front paws, located on a platform to allow the operator to access the periorbital area. The box size allowed for head and forepaw movements but prevented the animal from turning around inside it. One day before the first behavioral observations, mice were habituated to the apparatus. On the day of the experiment, after 20 min of adaptation inside the chamber, a series of seven von Frey filaments in logarithmic increments of force (0.08, 0.02, 0.04, 0.07, 0.16, 0.4, and 0.6 g) were applied to the periorbital area, perpendicular to the skin, with sufficient force to cause a slight buckling, and held for approximately 5 s to elicit a positive response. The response was considered positive by the following criteria: mouse vigorously stroked its face with the forepaw, head withdrawal from the stimulus, or head shaking. The stimulation was initiated with the 0.16 g filament. The absence of a response after 5 s led to the use of a filament with increased weight, whereas a positive response led to the use of a weaker (i.e., lighter) filament. A total of six measurements were collected for each mouse unless four consecutive positive or negative responses occurred. The 50% mechanical withdrawal threshold (expressed in g) was then calculated from these scores.

The mechanical threshold was evaluated in the periorbital region over the rostral portion of the eye (i.e., the area of the periorbital region facing the sphenoidal rostrum), initially as the basal threshold (before stress) and then on days 1, 3, 7, 10, and 14 post-stress induction. The PMA was also measured before ST or NST induction (basal), after ST or NST induction (7 days after), and 1 and 4 h after BIBN4096BS/vehicle treatment.

### 2.7 Open Field Test

The open-field test was used to analyze the locomotor activity, pain, and anxiety-like behaviors, such as grooming (s), rearing (s), sniffing (s), time into the peripheral zone, and crossings (number of times the line of a square is crossed with all four legs) (Antoniazzi et al., 2019). The apparatus was made of light-grey polyvinyl chloride (PVC) (50 × 50 × 25 cm). The test was performed over 30 min for 3 days (on days 1, 7, and 14) following stress induction and in the first hour after the treatment on day 7 post-stress. Illumination (about 40 lux) was provided by a light bulb hanging 60 cm above the apparatus. The temperature of the room was maintained at 22°C. The test was a recorded test using AnyMaze^®^ 7.0 software. Between each trial, the apparatus was cleaned with a 30% ethanol solution to avoid odor cues.

### 2.8 Sample Collection and Analysis

On day 7 post-stress induction or in the NST group, mice were anesthetized with ketamine-xylazine (100:20 i. p.) plus a maintenance dose of isoflurane (2.5%) and euthanized by cardiac puncture. The plasma was collected to determine the levels of corticosterone, cytokines, and CGRP.

### 2.9 Corticosterone Plasma Levels

The plasma was obtained on days 1 and 7 post-stress induction, after whole blood centrifugation at 3000 *g* at room temperature for 10 min and stored at -80°C until analysis. The corticosterone circulating level was measured using an ELISA Kit (Enzo Life Sciences, Farmingdale, New York, United States, Catalogue No. ADI-900-097), according to the product manual. Briefly, the samples were diluted according to protocol, and 100 µl of the sample or standard solutions were then incubated with the antibody using the plate provided, on a plate shaker at room temperature for 2 h. Afterward, the wells were emptied and washed, and then 200 µl pNpp substrate solution was added before incubating for 1 h without shaking. Finally, the plate was read at 405 nm using a microplate reader (SpectraMax I3; Molecular Devices, San Jose, California, United States).

### 2.10 CGRP Assay

Affinity sorbent (A19482, SPI Bio, Bertin Pharma) was used with a pool of different sources of plasma to prepare CGRP-free plasma. The samples were then analyzed using a commercially available CGRP kit (Catalogue No. A05482, SPI Bio, Bertin Pharma), following the manufacturer’s procedures. The concentration of CGRP was measured using Ellman’s Reagent to detect the enzymatic activity of the acetylcholinesterase, where the intensity of the yellow color formed was proportional to the amount of CGRP present in the sample (Castillo-Silva et al., 2019). The absorbance was read at 405 nm on SpectraMax I3 (Molecular Devices, San Jose, California, United States). The absorbance was read at 405 nm on a SpectraMax I3 (Molecular Devices, San Jose, California, United States). The absorbance values of the standards were used to plot a standard curve, from which the absorbance values of experimental samples were interpolated to determine their concentrations. This test was performed in mice plasma taken on the seventh-day post-stress induction.

### 2.11 Cytokine Level Determination in Plasma Samples

Cytokines in the plasma samples were measured on the seventh-day post-stress induction using a BD CBA Mouse Th1/Th2/Th17 Cytokine Kit (BD Bioscience, San Jose, CA, United States, Catalogue No. 560485). The kit was used for the simultaneous detection of mouse interferon-γ (IFN-γ), IL-2, IL-4, IL-6, IL-10, IL-17, and TNF-α in a single sample, according to the manufacturer’s instructions. Briefly, beads coated with seven specific capture antibodies were mixed. Subsequently, 50 μl mixed captured beads, 50 μl unknown serum sample or standard dilutions, and 50 μl phycoerythrin (PE) detection reagent were added consecutively to each assay tube and incubated for 2 h at room temperature in the dark. The samples were washed with 1 ml wash buffer for 5 min and centrifuged (200 g). The bead pellet was resuspended in a 300 μl buffer after discarding the supernatant. Samples were measured on the BD Accuri C6 flow cytometer, and data analysis was performed with FlowJo software. Individual cytokine concentrations were indicated by their fluorescent intensities. Cytokine standards were serially diluted to facilitate the construction of calibration curves, which were necessary to determine the protein concentrations (pg/ml) of the test samples.

### 2.12 Statistical Analysis

Data were presented as mean ± standard error of mean (S.E.M.). Data were statistically analyzed using parametric Student’s t-test, one-way ANOVA, repeated measures, and two-way mixed model analysis of Variance (ANOVA), according to the experimental protocol. The post hoc comparisons employed the Bonferroni criterion and *p* values less than 0.05 were considered significant. The maximal inhibition (Imax) was calculated using the following formula: 100 × (h post-treatment–mean of basal post-induction)/(basal post-induction mean–basal post-induction mean). Statistical analyses were performed using Graph Pad Prism 9.0 software.

## 3 Results

### 3.1 Unpredictable Sound Stress Evokes PMA, Hind Paw Mechanical Allodynia, and Grimacing Pain Behavior

We first determined the development of nociception post-stress on days 1, 3, 7, 10, and 14 and address whether there would be a sexually dimorphic effect. In this stress model, we observed an enhanced plasma corticosterone level on day 1 after stress induction, but not after 7 days of stress exposure (Supplementary Figure S1), as described before for this model using rats (Khasar et al., 2008; Khasar et al., 2009).

**FIGURE 2:**
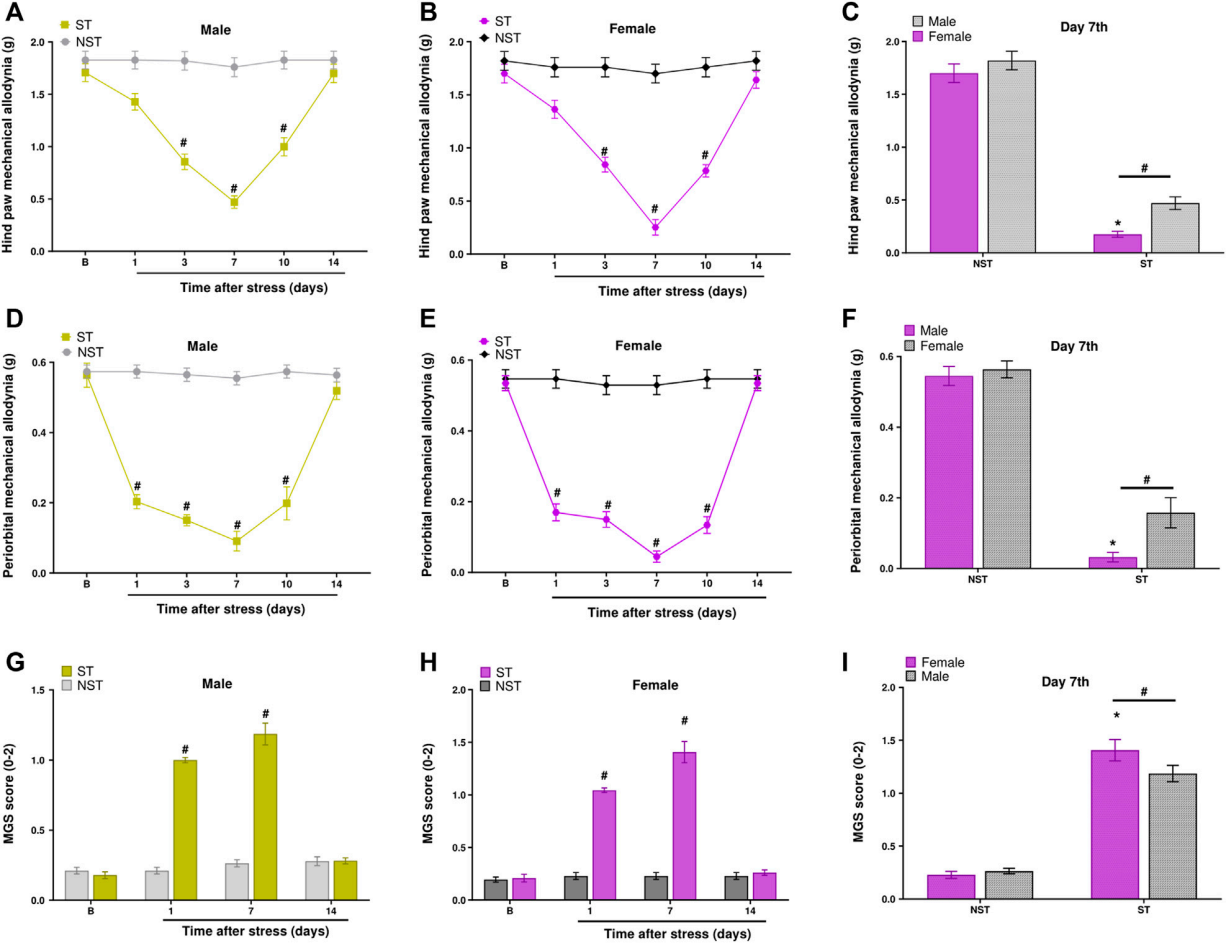
Unpredictable sound stress evokes periorbital mechanical allodynia (PMA), hind paw mechanical allodynia, and grimacing pain behavior in mice. Hind paw mechanical allodynia was measured in **(A)** male and **(B)** female mice at different time points after stress (ST group) or control (non-stressed group, NST). **(C)** Comparison of hind paw mechanical allodynia between male and female mice at day 7 after stress or in NST group. PMA was measured in **(D)** male and **(E)** female mice. **(F)** Comparison of PMA between male and female mice at day 7 after stress or in the NST group. Grimacing pain behavior was evaluated in **(G)** male and **(H)** female at different time points after stress (ST group) or control (non-stressed group, NST). **(I)** Comparison of grimacing pain behavior male and female mice at day 7 after stress or in NST group. Baseline measurements (described as B in the graph) were taken before induction. Spontaneous nociception was analyzed by the mouse grimace scale (MGS). Data are expressed as mean ± S.E.M. (*n* = 8). ^#^ *p* < 0.05 when compared to the NST group, and between days (1, 7, and 14 post-stress) comparing the ST group and NST group. *p < 0.05, when compared between the sexes within the ST group [repeated measures two-way ANOVA followed by Bonferroni’s *post hoc* test].

Male and female mice showed hind paw mechanical allodynia on days 3, 7, and 10 post-stress induction. Mice returned to baseline withdrawal thresholds 14 days post-stress induction (Figures 2A,B). Similarly, when verifying PMA, the allodynic effect of stress started on the first-day post-stress and was maintained until the 10th day in both sexes (Figures 2D,E). To measure spontaneous non evoked pain in these animals, we assessed grimace, observing that both males and females in the ST group had grimacing pain behavior on days 1 and 7 post-stress compared to NST mice (Figures 2G,H).

Thus, we verified that the nociceptive peak was on the seventh-day post-stress for PMA, hind paw mechanical allodynia, and grimacing pain behavior. Interestingly, at the nociceptive peak, sexual dimorphism was observed for hind paw mechanical allodynia, PMA, and grimacing pain behavior. These data showed that females had higher nociception compared to males in the ST group on the seventh-day post-stress induction (Figures 2C,F,I); however, male and female mice had a similar development of nociception on days 1, 3, 10, and 14 post-stress induction (Supplementary Figure S2).

### 3.2 Induction of Anxiety-Like Symptoms and Decrease in Exploratory Activity After Unpredictable Sound Stress

**FIGURE 3:**
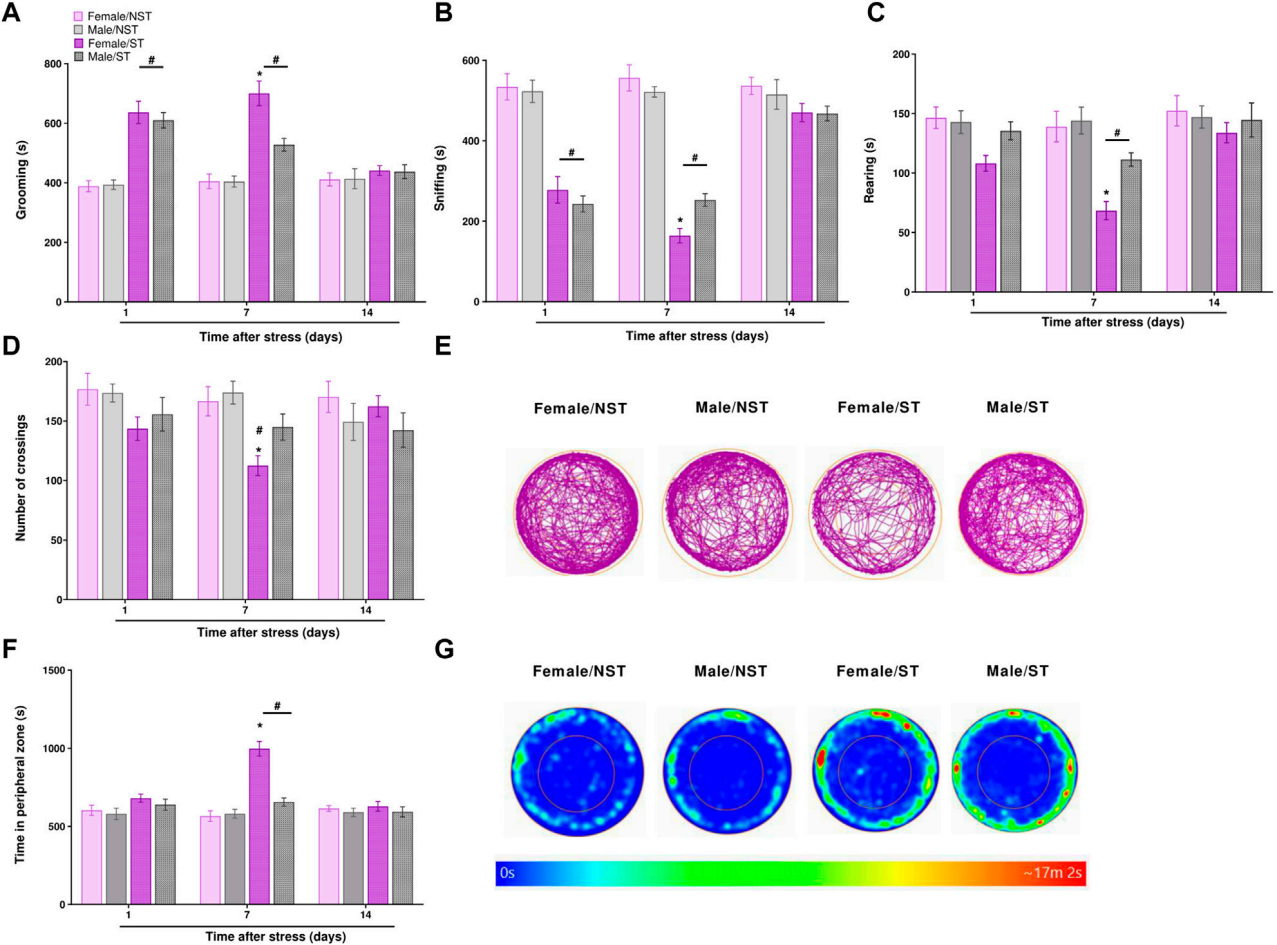
Anxiety-like symptoms induction and decrease exploratory activity after unpredictable sound stress induction. Time spent **(A)** grooming **(B)** sniffing **(C)** rearing **(D)** the number of crossings **(E)** representative image of crossings represented by track plot **(F)** time in the peripheral zone were observed in the open field test on days 1, 7, and 14 post-stress (ST group) or in non-stressedgroup (NST). **(G)** Representative image of time in the peripheral zone represented by heat map. Data are expressed as mean ± S.E.M. (n = 8). *p < 0.05, when compared between the sexes within the ST group. ^#^ *p* < 0.05 when compared to the NST group. [Repeated measures two-way ANOVA followed by Bonferroni’s p’ost hoc test].

The open-field test was evaluated on days 1, 7, and 14 post-stress induction or in the NST group, measuring grooming (s), rearing (s), sniffing (s), time into the peripheral zone, and the number of crossings. The ST group spent more time grooming on both days 1 and 7 post-stress compared to the NST group, but not on day 14 (Figure 3A); on day 7, the ST females spent more time in grooming behavior compared to male ST mice (Figure 3A). The ST group spent significantly less time sniffing, both on days 1 and 7 (Figure 3B), with female mice showing a higher reduction in sniffing time compared to male ST mice on day 7 post-stress induction (Figure 3B). Male and female ST mice also spent less time rearing on day 7 post-stress induction compared to the NST group, and female ST mice showed a lower rearing time on day 7 compared to male ST mice (Figure 3C). We only perceived a reduction in the number of crossings on day 7 in ST female mice compared to NST female and ST male mice (Figure 3D,E). Then, we demonstrated that ST mice had more anxiety-like behavior than the NST group on the seventh-day post-stress induction, but no difference was detected on days 1 and 14 (Figures 3F,G). Also, when comparing the sex effect in the ST group, we evidenced that ST females presented anxiety-like behavior compared to ST males on day seventh after stress (Figures 3F,G). No significant difference was seen between groups in locomotor activity assessed by distance traveled (Supplementary Figure S3A). Thus, the induction of anxiety-like symptoms and decrease in exploratory activity were mainly detected on day 7 post-stress induction, and this day was therefore chosen to measure the antinociceptive effect of CGRP antagonist treatment and the analysis of plasma levels of pro-inflammatory cytokines and CGRP.

### 3.3 Unpredictable Sound Stress Induces an Increase in IL-6, TNF-α, and CGRP Levels in Plasma

**FIGURE 4:**
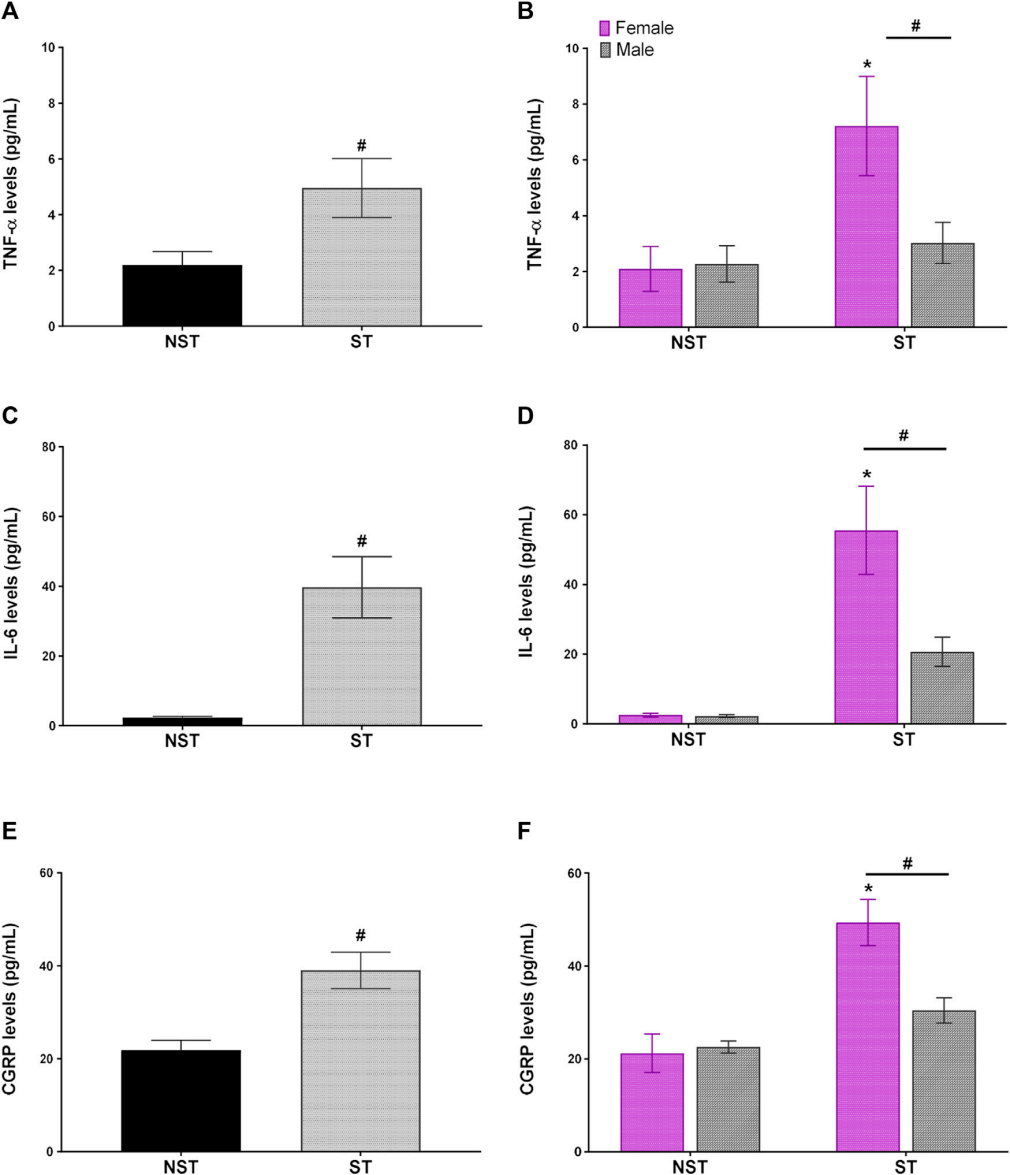
Unpredictable sound stress induces an increase in IL-6 (Interleukin-6), TNF-α TNF-α (tumor necrosis factor-alpha), and CGRP calcitonin gene-related peptide levels in plasma. Plasmatic levels of **(A,B)** TNF-α **(C,D)** IL-6 (Interleukin-6), and **(E,F)** CGRP detected at 7 days after stress (ST group) or in non-stressed group (NST). Data are expressed as mean ± S.E.M. (n = 12) in the graphs **(A,C,D)** and *n* = 6 in the graphs **(B,D,F)** ^#^ *p* < 0.05, when compared to NST group in **(A,C,E)** [parametric Student’s *t*-test], and **(B,D,F)** [Two-way ANOVA followed by Bonferroni’s *post hoc* test]. *p < 0.05 when compared between the sexes within the ST group.

Unpredictable sound stress induced high levels of circulating pro-inflammatory cytokine, such as IL-6 and TNF-α, in plasma compared to NST animals on day 7 post-stress (Figures 4A,C). Moreover, in the same way, the levels of CGRP in plasma were higher in the ST group than in the NST group (Figure 4E). When comparing the effect of sexual dimorphism in the ST group, the females presented higher levels of TNF-α, IL-6, and CGRP than their male counterparts (Figures 4B,D,F). Nevertheless, there was no difference in the plasma levels of IFN-γ, IL-2, IL-4, IL-10, and IL-17 between the groups (Supplementary Figure S4).

### 3.4 The CGRP Antagonist Reduced the Migraine-Like Behaviors Induced by the Unpredictable Stress Model in Mice

**FIGURE 5:**
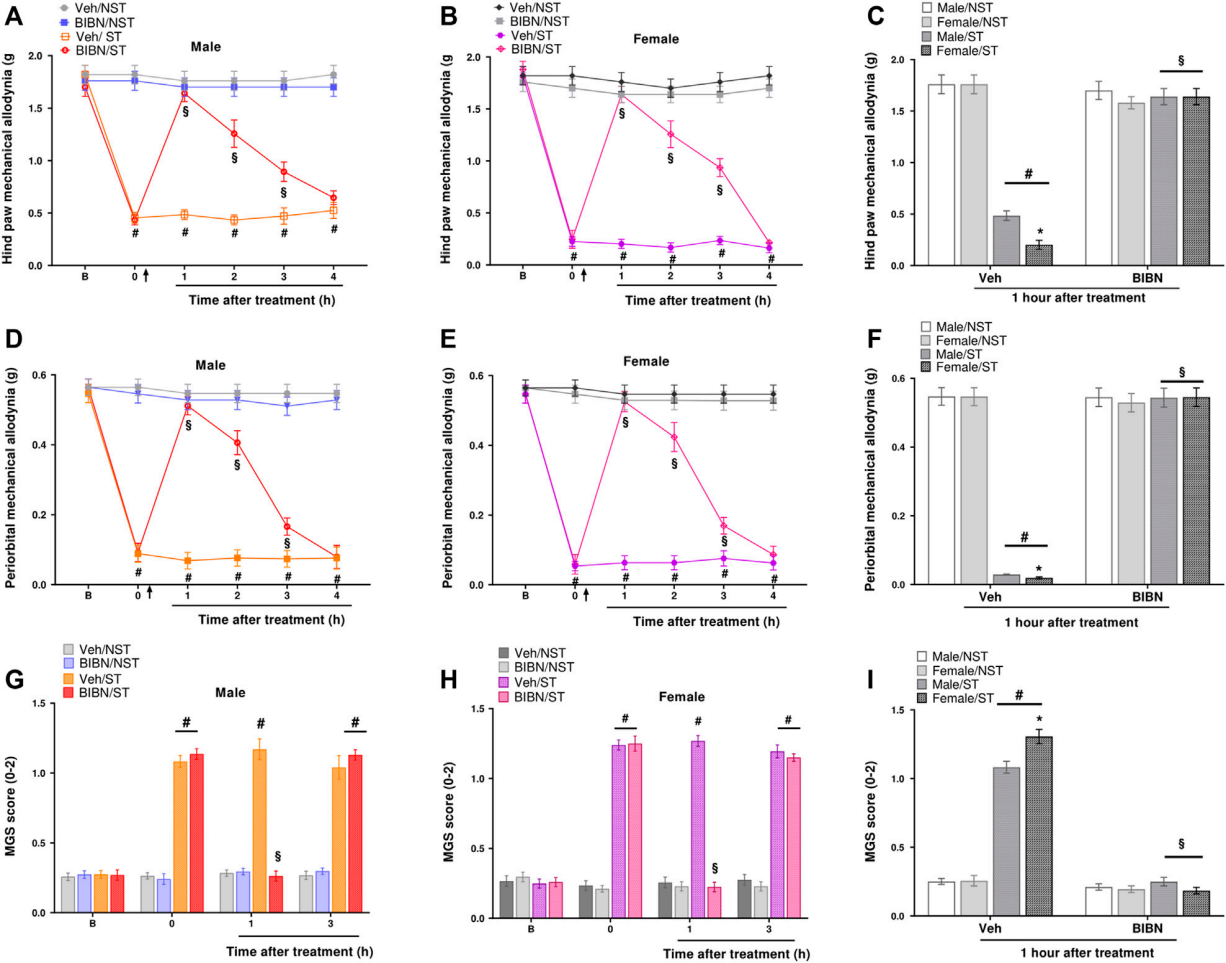
CGRP antagonist reduced migraine-like behaviors induced by unpredictable stress model in mice. BIBN4096BS (olcegepant, 100 mg/kg) intraperitoneal (i.p.) administration reduced the **(A,B)** hind paw mechanical allodynia **(D,E)** periorbital mechanical allodynia (PMA), and **(G,H)** grimacing pain behavior in male and female stressed (ST) mice when compared to vehicle (Veh) treated group. Antinociceptive effect of BIBN4096BS at 1 h after treatment for **(C)** hind paw mechanical allodynia **(F)** PMA, and **(I)** grimacing pain behavior. Baseline measurements (described as B in the graph) were observed before induction. Time 0 represents the measures taken 7 days after ST or in the NST group. The arrow represents the treatment or veh injection. Spontaneous nociception was analyzed by the mouse grimace scale (MGS). Data are expressed as mean ± S.E.M. (*n* = 8). *p < 0.05, when compared vehicle/stressed group (ST) or between the sexes within the ST group. ^#^ *p* < 0.05 when compared to the NST group ^§^ *p* < 0.05 when compared to the vehicle (Veh) treated group [repeated measures two-way ANOVA followed by Bonferroni’s *post hoc* test].

To evaluate the CGRP role in migraine-like behaviors induced by sound stress, we administered a CGRP antagonist (BIBN4096BS) by the i. p. route (100 mg/kg) on the seventh-day post-stress induction. We observed that this compound had a robust effect on reducing hind paw mechanical allodynia induced by stress, 1–3 h post-dose in male and female mice in the ST group (Figures 5A,B). The Imax was 87 ± 6% and 88 ± 5% for male and female mice in the ST group, respectively, 1 h after BIBN4096BS injection for hind paw mechanical allodynia (Figure 5C). This compound also showed a periorbital antiallodynic effect 1–3 h after treatment (Figures 5D,E), with an Imax of 92 ± 5% and 93 ± 5% for male and female mice in the ST group, respectively, 1 h after BIBN4096BS treatment for PMA (Figure 5F). In an analogous way, we demonstrated that BIBN4096BS significantly reduced the grimacing pain behavior in male and female mice in the ST group, 1 h after treatment, compared to the vehicle-treated group (Figures 5G,H). Thus, 1 h after treatment, BIBN4096BS showed similar antinociceptive effects in female and male ST mice (Figures 5C,F,I). Also, after 2 and 3 h, BIBN4096BS showed similar antinociceptive effects for hind paw mechanical allodynia and PMA in female and male ST mice (Supplementary Figure S5).

### 3.5 The CGRP Antagonist Reverted the Anxiety-Like Symptoms and Increased Exploratory Activity Induced by the Unpredictable Sound Stress

**FIGURE 6:**
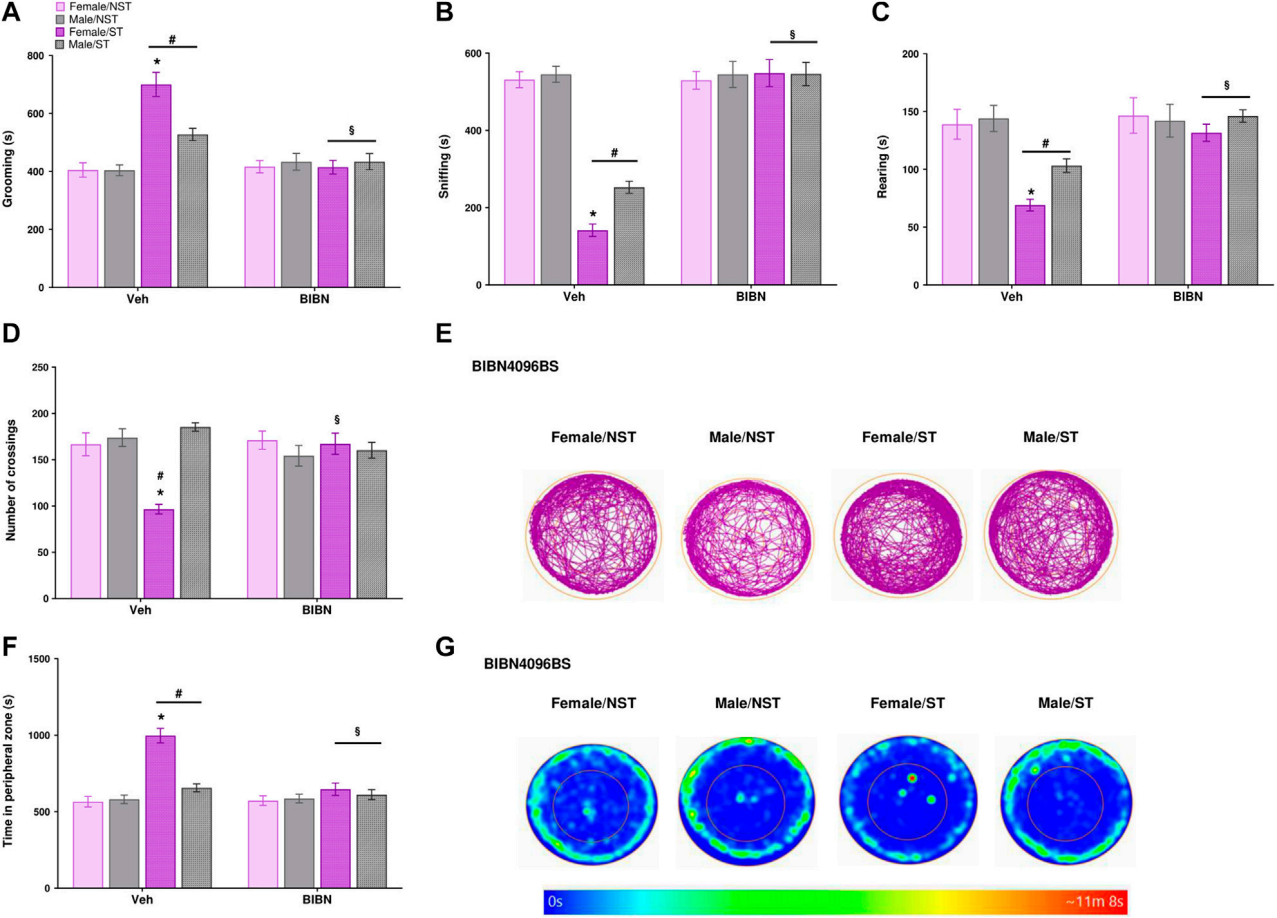
CGRP antagonist reverts the anxiety-like symptoms and increased exploratory activity after unpredictable sound stress. BIBN4096BS (olcegepant, 100 mg/kg) intraperitoneal (i.p.) administration reverted the alterations of different behavioral parameters: Time spent **(A)** grooming **(B)** sniffing **(C)** rearing **(D)** the number of crossings **(E)** representative image of crossings represented by track plot **(F)** time in the peripheral zone were observed in the open field test at day 7 post-stress (ST group) or in non-stressed group (NST) 1 hour-post BIBN4096BS administration. **(G)** Representative image of time in the peripheral zone represented by heat map. Data are expressed as mean ± S.E.M. (*n* = 8). *p < 0.05, when compared between the sexes within the ST group. ^#^ *p* < 0.05 when compared to the NST group. ^§^ *p* < 0.05 when compared to the vehicle (Veh) treated group [repeated measures two-way ANOVA followed by Bonferroni’s *post hoc* test].

The effect of BIBN4096BS treatment was evaluated 1 h after drug administration using the open field test. We perceived that the drug was able to reverse the alterations observed post-stress induction, reverting the grooming parameter in male and female ST mice compared to the vehicle-treated group (Figure 6A) and reverting the reduced exploratory behavior, expressed as the sniffing time, in ST mice compared to the vehicle-treated group (Figure 6B). BIBN4096BS was also able to increase the rearing behavior (Figure 6C) in ST mice compared to the vehicle group (Figure 6D). Regarding the number of crossings, the treatment reverted the stress effect in female mice (Figures 6D,E) compared to females of the vehicle group. Finally, the treatment reduced the time spent in the peripheral zone in the ST group when compared to the vehicle group (Figures 6F,G), but the treatment did not affect the distance traveled (Supplementary Figure S3B). The treatment showed no significant difference in the effects (anxiety-like symptoms and exploratory activity) of BIBN4096BS for male and female ST mice (Figure 6).

## 4 Discussion

Migraine has a higher prevalence in females and negatively affects the quality of life of patients (Feigin et al., 2020). Stress is a common trigger reported in migraine patients, but the underlying mechanisms are poorly understood (Kelman, 2007; Viero et al., 2022a). Indeed, different types of stress, such as repetitive restrain stress paradigm, social defeat stress, chronic variable stress, and early life stress have been used as models of migraine-like behavior in animals (Kaufmann and Brennan, 2018; Avona et al., 2020a; Eller et al., 2021). In this sense, the unpredictable sound stress was used as a model to induce painful hypersensitivity in rats (Khasar et al., 2009; Dina et al., 2011). Sound stress also represents a type of stress that normally occurs in humans (Ishikawa et al., 2019; Yamashita et al., 2021). However, nociceptive behaviors have not been evaluated in this stress model in mice, and this model has not yet been characterized as a model of migraine. Thus, we have characterized migraine-like behaviors in a stress model mediated by unpredictable sound in male and female mice.

In this sense, previous studies have shown that a significant proportion of individuals with migraine have experienced cutaneous allodynia and spontaneous pain during episodes of headache (Karsan and Goadsby, 2018). Different models of migraine evaluate the development of periorbital and hind paw allodynia, and grimacing pain behavior (Kaufmann and Brennan, 2018; Avona et al., 2020a). Here, we detected these nociceptive parameters after the induction of unpredictable sound stress, with a nociceptive peak at day 7 post-stress induction. Recently, using a model of repeated restrain stress in mice, Avona and others (Avona et al., 2020a) showed PMA and grimacing pain behavior in mice. Similarly, we also observed that nociception was not detected 14 days after stress induction, either in terms of mechanical allodynia or grimacing pain behavior. Thus, we have characterized a new model of migraine-like pain induced by unpredictable sound stress. We demonstrated an enhanced plasma corticosterone level on day 1 after stress induction, which corroborates the previous studies utilizing the same model in rats (Strausbaugh et al., 2003; Khasar et al., 2008). This is an interesting model because migraine patients are more conscious of sound, and studies have described that sound could be a stress factor to induce migraine pain attacks (Ishikawa et al., 2019; Yamashita et al., 2021).

Avona and others also demonstrated that stress induction in mice elicits migraine-like behaviors, causing a sensitization in all the studied animals (Avona et al., 2020a). On the other hand, it is known in the literature that stress does not induce a migraine attack in all patients, and, in non-migraineurs, stress does not produce migraine (Stubberud et al., 2021). However, other animal models of headache and migraine developed in mice and rats also induced nociception in all the animals, as demonstrated by prior surveys (Dina et al., 2011; Avona et al., 2020b; Eller et al., 2021).

While head pain is a typical feature of migraine attacks, it also often presents with cutaneous hypersensitivity in the rest of the body (Goadsby et al., 2017). Stress also has a relationship with the development of allodynia in other primary headache disorders such as tension-type headache (Cathcart et al., 2008). Also, cutaneous allodynia reflects central sensitization that might be occurring clinically. However, the mechanisms by which extracranial allodynia occurs remain largely unknown. Additionally, it is already described that an animal model of migraine headache that produced reproducible and quantifiable cutaneous allodynia could be utilized to mechanistically study the development and maintenance of migraine-related pain (Edelmayer et al., 2012).

Migraine is also associated with psychiatric disorders, such as anxiety (Karimi et al., 2021). Some previous studies indicate that nociception in migraine-like models could be accompanied by exploratory behavior alteration and anxiety-like symptoms (Araya et al., 2020; Taheri et al., 2020). In this respect, we also detected that stress induction decreased the time of different exploratory behaviors, such as rearing and sniffing, and the number of crossings. In addition, stress was correlated with an increased grooming time and permanence in the peripheral zone. In previous studies using models of migraine, a reduction in rearing behavior was accepted as being indicative of spontaneous pain and a decrease in feeding and exploratory behavior (Edelmayer et al., 2012), an increase in facial grooming (Filiz et al., 2019) was also correlated to migraine-like pain. After exposure to repeated stress, mice stayed longer in the peripheral zone than the control mice (Kaufmann and Brennan, 2018; Hwang et al., 2020), which is a behavior parameter widely used for assessing anxiety (Seibenhener and Wooten, 2015). Additionally, in the migraine-like model induced by nitroglycerin, authors verified increased anxiety-like symptoms in the open field test in rats (Taheri et al., 2020). Our study also presented other aspects related to migraine-like pain, such as anxiety-like symptoms and a decrease in exploratory behavior, which are relevant aspects of a model of migraine (Kaufmann and Brennan, 2018; Avona et al., 2020a; Hwang et al., 2020).

Migraine affects three times more women than men, but the mechanisms underlying this dimorphism are not known (Vetvik and MacGregor, 2017; Avona et al., 2020a). Some studies have shown a sexual dimorphism in migraine-like models, with female rodents showing higher nociceptive and anxiety-like responses (Araya et al., 2020). Recently, using a migraine model induced by nitroglycerin i. p. injection, a sexual dimorphic effect was detected, in which females had higher nociception than males (Alarcón-Alarc ón et al., 2021). However, most of the studies using models of migraine have not evaluated sexual differences, and male mice or rats are normally tested (Khasar et al., 2009; Taheri et al., 2020). Previous studies utilizing sound stress have demonstrated hind paw nociception but have only performed the nociceptive tests in male rats (Crettaz et al., 2013). Only one study evaluated sexual differences in mice after stress induction (repeated restrain stress) but did not find any difference in PMA and grimacing pain behavior (Avona et al., 2020a). Nevertheless, we have demonstrated that unpredictable sound stress induces higher nociceptive and anxiety-like parameters in females than in ST males, and it is likely that the differences detected could be induced by the stress model used.

Besides, TNF-α and IL-6 showed that increased plasma levels have already been demonstrated in migraine patients compared to healthy controls (Oliveira et al., 2017; Kursun et al., 2021). Similarly, in rodents, pro-inflammatory cytokines were described as inductors of primary afferent nociceptors sensitization (Khasar et al., 2008; Kursun et al., 2021) and high CGRP plasma levels have already been demonstrated in external jugular venous blood, saliva, and cerebrospinal fluid of migraine patients during an attack (Alpuente et al., 2020). The migraine animal model also induces an increase in circulating CGRP levels in plasma (Cohen et al., 2021; Sun et al., 2021) and cerebrospinal fluid of the nestin/hRAMP1 transgenic mice (Recober et al., 2009). Additionally, CGRP may trigger the release of pro-inflammatory cytokines (Ray et al., 2021), particularly IL-6 and TNF-α, which are also increased in the plasm of migraine patients (Yücel et al., 2016; Conti et al., 2019).

In accordance with these findings, we have demonstrated that unpredictable sound stress induced elevated levels of circulating of IL-6, TNF-α, and CGRP in male and female mice. Acute stress models, including restrain and social isolation, caused high plasma levels of IL-6 (Qing et al., 2020; Shi et al., 2022). However, no model of stress-inducing migraine-like behavior had done this measure before (Kaufmann and Brennan, 2018; Avona et al., 2020a; Eller et al., 2021). The injection of IL-6 (dura mater and cisterna magna) and CGRP (dura mater and intraganglionar injection) induced nociception (Avona et al., 2019a; Araya et al., 2020), and dural meningeal nociceptors were also sensitized by TNF-α application (Zhang et al., 2011). Additionally, unpredictable sound stress model-induced muscle nociception could be reduced by antisense injection to IL-6 or TNF-α receptors (Dina et al., 2011). Seeking to elucidate the sexual dimorphism presented in nociceptive parameters, the ST females presented higher levels of TNF-α, IL-6, and CGRP levels than male ST mice. Our results corroborate the study of Avona and others, in which the sexually dimorphic effect of CGRP female-specific hypersensitivity responses was seen in mice, where increased grimace responses were also observed (Avona et al., 2019b). In fact, estrogen has been shown to regulate the release of CGRP (Stucky et al., 2011; Pota et al., 2017). The data presented are innovative and, to our knowledge, have not previously been assessed in migraine models. Thus, this model can be explored in future preclinical studies and research with migraine patients.

Recent reports indicate that mechanisms of pain may differ between the sexes, and a potential role for spinal prolactin has been implicated in the production of IL-6 induced hind paw allodynia (Mapplebeck et al., 2017; Al-Hassany et al., 2020) and a co-injection of prolactin with IL-6 similarly increases hind paw hypersensitivity in female mice (Patil et al., 2019). Furthermore, several studies have indicated that fluctuations of ovarian steroid hormone (mainly estrogen) levels modulate CGRP in the trigeminovascular system during different reproductive milestone migraine and suggest that female-specific mechanisms downstream of CGRP receptor activation contribute to the higher prevalence of migraine in women (Labastida-Ramírez et al., 2019).

Recent clinical studies have further confirmed a protagonist of CGRP in migraine due to the use of CGRP signaling inhibitors, such as antagonists and monoclonal antibodies to CGRP receptor (Halker Singh et al., 2019). Here, we have demonstrated that a CGRP receptor antagonist had an antinociceptive and anxiolytic-like effect and reverted the reduced exploratory behavior alterations, showing similar efficacy in male and female ST mice. Olcegepant (BIBN4096BS) was also used to reduce the nociception observed in other models of migraine (Dalenogare et al., 2021). Using a model of repetitive retrain stress, the authors also showed that CGRP signaling may be involved in the migraine-like behaviors detected in this model of stress. In this study, the CGRP monoclonal antibody ALD405 reduced nociception, mainly in female mice, but the authors used the priming effect of a nitric oxide donor (Avona et al., 2020a). In migraine patients, olcegepant can reduce migraine pain in both male and female patients (Xu and Sun, 2019). Besides, intracerebroventricular CGRP infusions are reported to be involved in various behaviors suggestive of anxiety (Clemow et al., 2020). In this sense, the CGRP antagonist showed antidepressant-like effects in stressed mice, and CGRP antagonism has been shown to suppress anxiety-like behaviors in rats (Filiz et al., 2019).

In fact, more experiments are needed for a solid conclusion regarding the difference between males and females and the effect of the antagonist BIBN4096BS on cytokines and CGRP levels. Nonetheless, this is an exploratory study in which we preferred to perform the acute treatment to verify if the drug would have an antinociceptive effect or alter the anxiety-like and locomotor parameters. To elucidate the mechanism of migraine triggered by the unpredictable sound, later it is intended to do the BIBN4096BS treatment for more days, as a chronic treatment. Also, in a future study, it will be interesting to verify if IL-6, TNF-α, and CGRP levels are affected by BIBN4096BS treatment. Besides, interestingly, Avona and others (Avona et al., 2020a) showed that after repeated restraint stress, mice have PMA and grimacing pain behavior, but after 14 days of stress, induction nociception was not detected. Then, they injected IL-6, inflammatory soup, and nitroglycerin, and a hyperalgesic priming (or latent sensitization) was observed. Thus, in future studies, it will be interesting to verify if the same type of effect occurs with the unpredictable sound stress model.

Our data show that the unpredictable sound stress model in mice causes periorbital/hind paw mechanical allodynia and grimacing pain behavior. In this model, we also detected anxiety-like symptoms and decreased exploratory activity. The nociception, anxiety-like behavior, and exploratory activity alterations detected in this model showed sexual dimorphism; thus, female mice presented higher levels of hind paw mechanical allodynia, PMA, grimacing pain behavior, and anxiety-like symptoms after stress induction. In addition, we demonstrated that ST female mice presented lower levels of exploratory behavior than males of the same group, and the plasma levels of inflammatory cytokines (IL-6 and TNF-α) and CGRP were increased in ST mice, particularly in the females. The CGRP antagonist also caused an antinociceptive effect in male and female ST mice, reducing the anxiety-like behavior and associated exploratory alterations related to this model. Therefore, these data support the use of this stress priming model in the study of the mechanisms, by which stress contributes to migraine-related pain and anxiety-like symptoms.

## Data Availability

The raw data supporting the conclusion of this article will be made available by the authors, without undue reservation.
