# Supplementary figures and images for: Unpredictable Sound Stress Model Causes Migraine-Like Behaviors in Mice With Sexual Dimorphism
Source: Front Pharmacol. 2022 Jun 16;13:911105. doi: 10.3389/fphar.2022.911105 (PMC9243578; doi:10.3389/fphar.2022.911105)

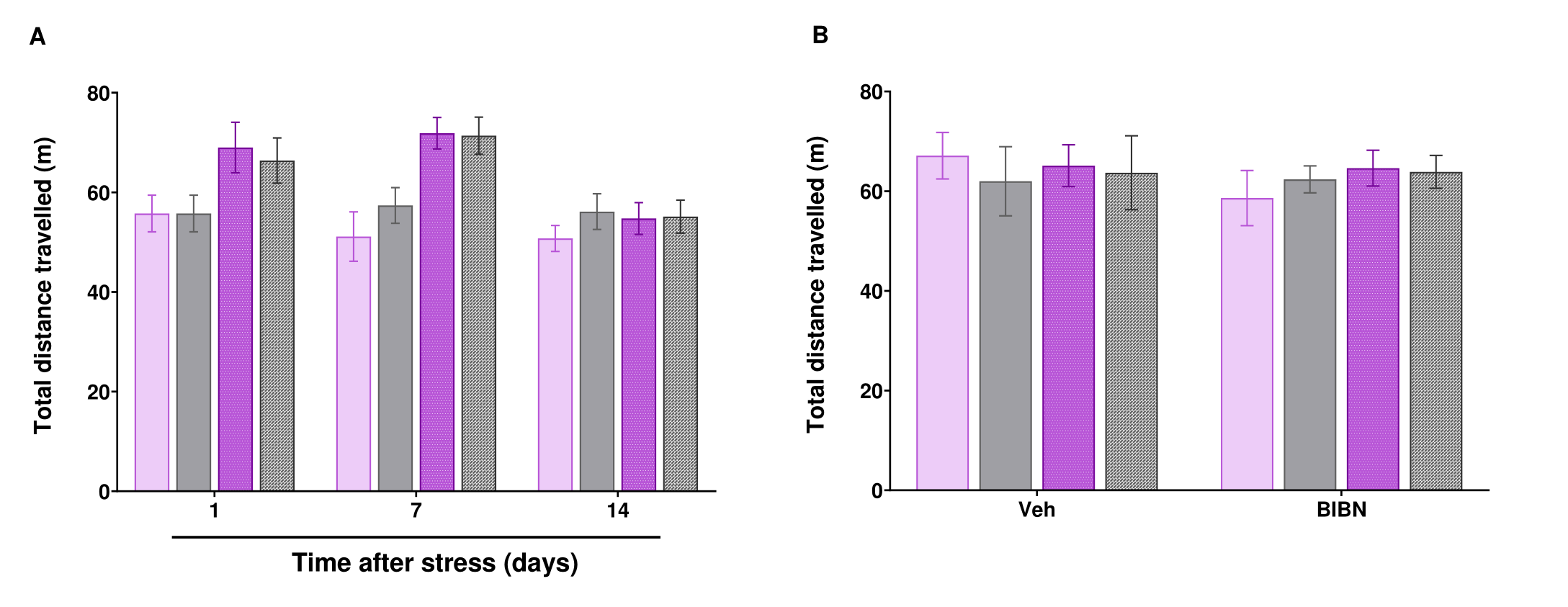

Supplement: Supplementary file 1 [file Image3.TIFF]

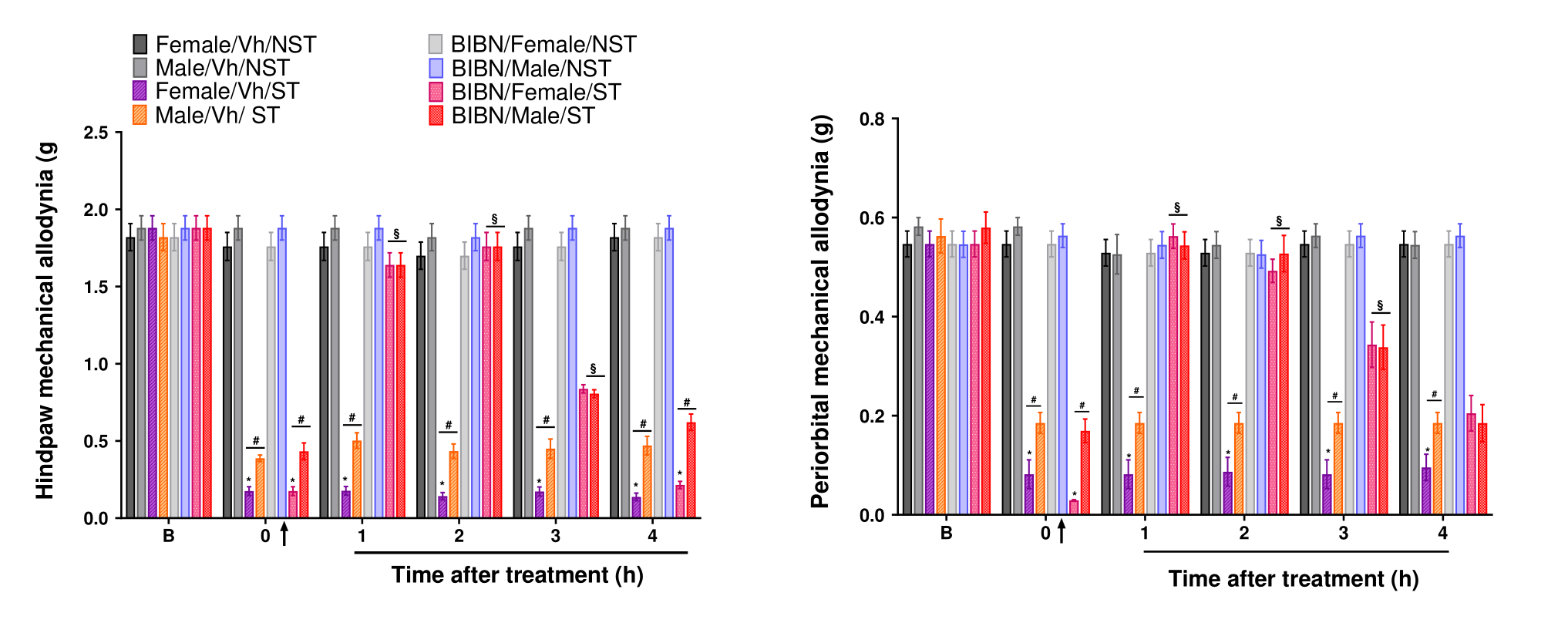

Supplement: Supplementary file 3 [file Image5.TIFF]

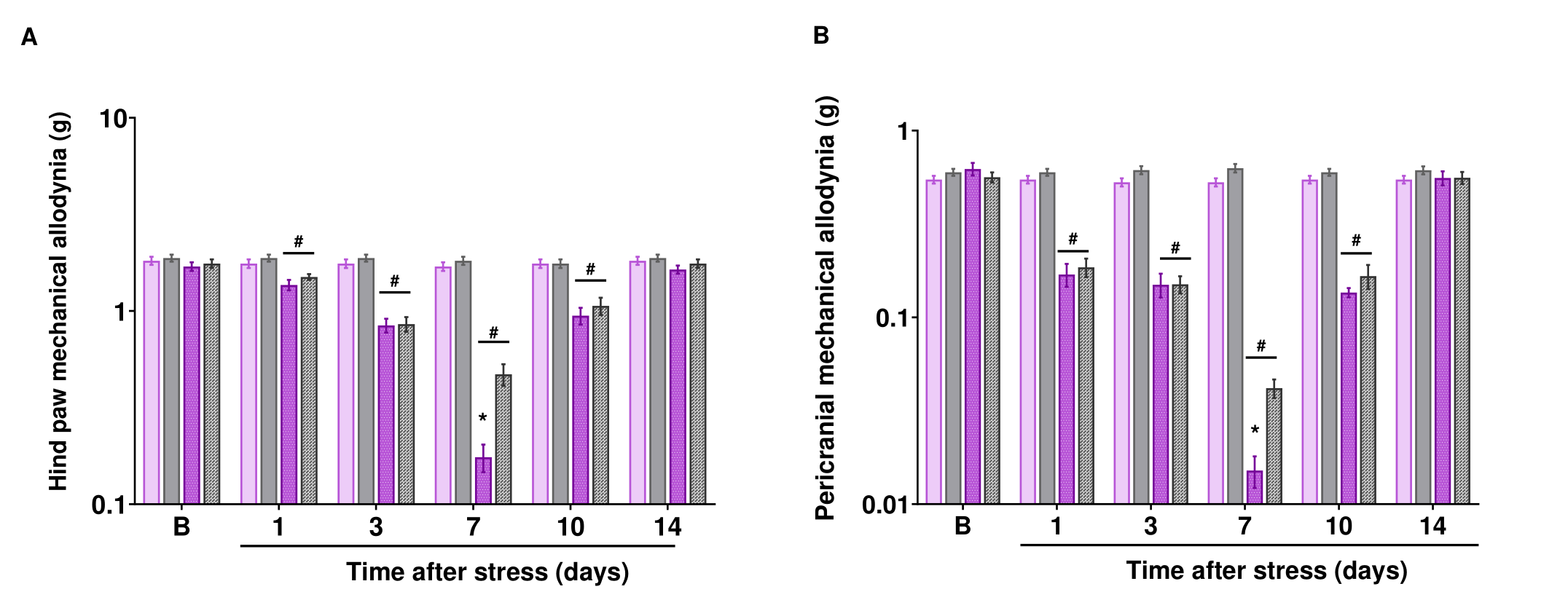

Supplement: Supplementary file 4 [file Image2.TIFF]

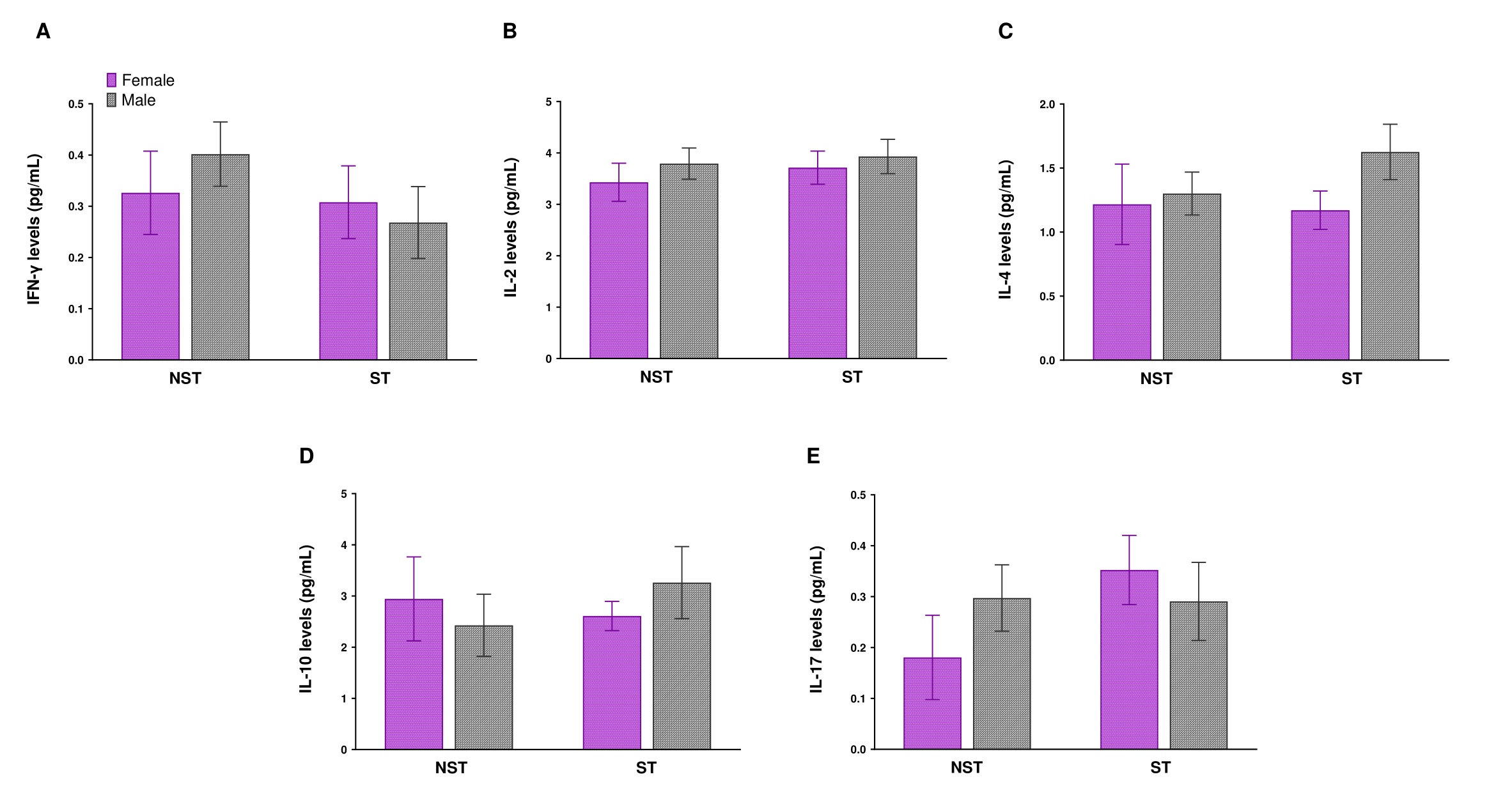

Supplement: Supplementary file 5 [file Image4.TIFF]
